# Knowledge-map analysis of percutaneous nephrolithotomy (PNL) for urolithiasis

**DOI:** 10.1007/s00240-023-01406-w

**Published:** 2023-01-20

**Authors:** Junhui Hou, Zongwei Lv, Yuan Wang, Xia Wang, Yibing Wang, Kefeng Wang

**Affiliations:** 1 Department of Urology, Shengjing Hospital of China Medical University, #36 Sanhao Street, Heping District, Shenyang, 110004 Liaoning China; 2 Department of General Surgery, Shengjing Hospital of China Medical University, #36 Sanhao Street, Heping District, Shenyang, 110004 Liaoning China

**Keywords:** Percutaneous nephrolithotomy, Urolithiasis, Bibliometrics, VOSviewer, Co-citation

## Abstract

Percutaneous nephrolithotomy (PNL) has been used in the treatment of urolithiasis for more than 20 years. However, bibliometric analysis of the global use of PNL for urolithiasis is rare. We retrieved the literatures on PNL and urolithiasis from Web of science core collection database. VOSviewer was used to analyze keywords, citations, publications, co-authorship, themes, and trend topics. A total of 3103 articles were analyzed, most of which were original ones. The most common keywords were “percutaneous nephrology” and “urolithiasis”, both of which were closely related to “ureteroscopy”. *Journal of Urology* and Zeng Guohua from the First Affiliated Hospital of Guangzhou Medical University were the most published journal and author in this field. The most productive country was the United States, and its closest partners were Canada, China, and Italy*.* The five hot topics were the specific application methods and means, risk factors of urolithiasis, the development of treatment technology of urolithiasis, the characteristics, composition, and properties of stones, and the evaluation of curative effect*.* This study aimed to provide a new perspective for PNL treatment of urolithiasis and provided valuable information for urologic researchers to understand their research hotspots, cooperative institutions, and research frontiers.

## Introduction

Urolithiasis is a common urological disorder, which includes kidney stones, ureteral stones, bladder stones, and urinary tract stones. The incidence of urolithiasis ranges from 7–13% in North America, 5–9% in Europe, and 1–9% in Asia [1]. The main symptoms of urolithiasis are sudden onset of low back pain and hematuria. Urolithiasis may lead to serious consequences without timely treatment, such as hydronephrosis, uremia, infection and abscess, acute kidney injury, and renal failure, threatening patients’ health and life [2–9]. However, it is reported that the prevalence and incidence of urolithiasis are increasing worldwide, which is threatening human’s civilization and health [10, 11].

There are three treatments for urolithiasis: extracorporeal shock wave lithotripsy (ESWL), ureteroscopy, and percutaneous nephrolithotomy (PNL) [12]. Among them, PNL shows the highest stone clearance rate in the surgical treatment of urolithiasis according to the American Urological Association guidelines. In contrast, ESWL is the least efficient [13, 14]. For this reason, PNL is widely studied and used on the treatment of urolithiasis. Despite the high efficiency of PNL, there is no bibliometric analysis of the publication trend of PNL in the treatment of urolithiasis through visual analysis.

Bibliometrics is a statistical method for quantitative analysis of research papers related to a topic through mathematical methods [15–17]. Bibliometrics can evaluate the quality of research, find the most valuable topics in the field, and identify research hotspots. Bibliometric analysis can also find the relationship between studies, predict the future research directions, provide new ideas for researchers, and greatly improve the efficiency of scientific research. The Web of science (WOS) online database provides the data for bibliometric analysis, and the data will be imported into softwares such as VOSviewer for further analysis.

The purpose of this study was to use bibliometric method to analyze a large number of studies on PNL in the treatment of urolithiasis from a macro perspective. By understanding the characteristics of bibliometrics analysis, we provided research directions and specific tasks that may belong to related research fields.

## Materials and methods

### Database and search methodology

On February 17th, 2022, WOS core database was used to retrieve literature on PNL and urolithiasis. WOS core database was an online subscription-based scientific citation index service provided by Carivate Analytics. It included articles from a variety of fields, providing retrieval and analysis capabilities. In addition, it can export data for further analysis.

The scope of the database in this study was limited to SCI-expanded, SSCI, CCR-expanded, and IC. The document type was set to “all types”, the language was set to “all languages”, and the time was set from 2000 to 2022.

In the advanced search field, we used the field tag “TS = topic” to boraden our search to find the keywords we need in the title, abstract, and keywords. The following queries were used: (TS = (percutaneous nephrolithotomy) OR TS = (PNL)) AND (TS = (urolithiasis) OR TS = (nephrolithiasis) OR TS = (ureterolithiasis) OR TS = (urinary calculi) OR TS = (kidney calculi) OR TS = (ureteral calculi) OR TS = (urinary bladder calculi) OR TS = (ureteral calculi) OR TS = (kidney stone) OR TS = (urostone) OR TS = (renal stone) OR TS = (urinary stone) OR TS = (ureteral stone) OR TS = (urinary bladder stone)).

### Data extraction and analysis

The bibliometric data were searched and downloaded from the WOS core database. We then imported the data into VOSviewer for further analysis and network creation. The software can be downloaded from the internet and used to analyze keywords, citations and publications, co-authorship, themes and trend topics of this study.

## Results

### Bibliometric analysis of publication output

**Fig. 1:**
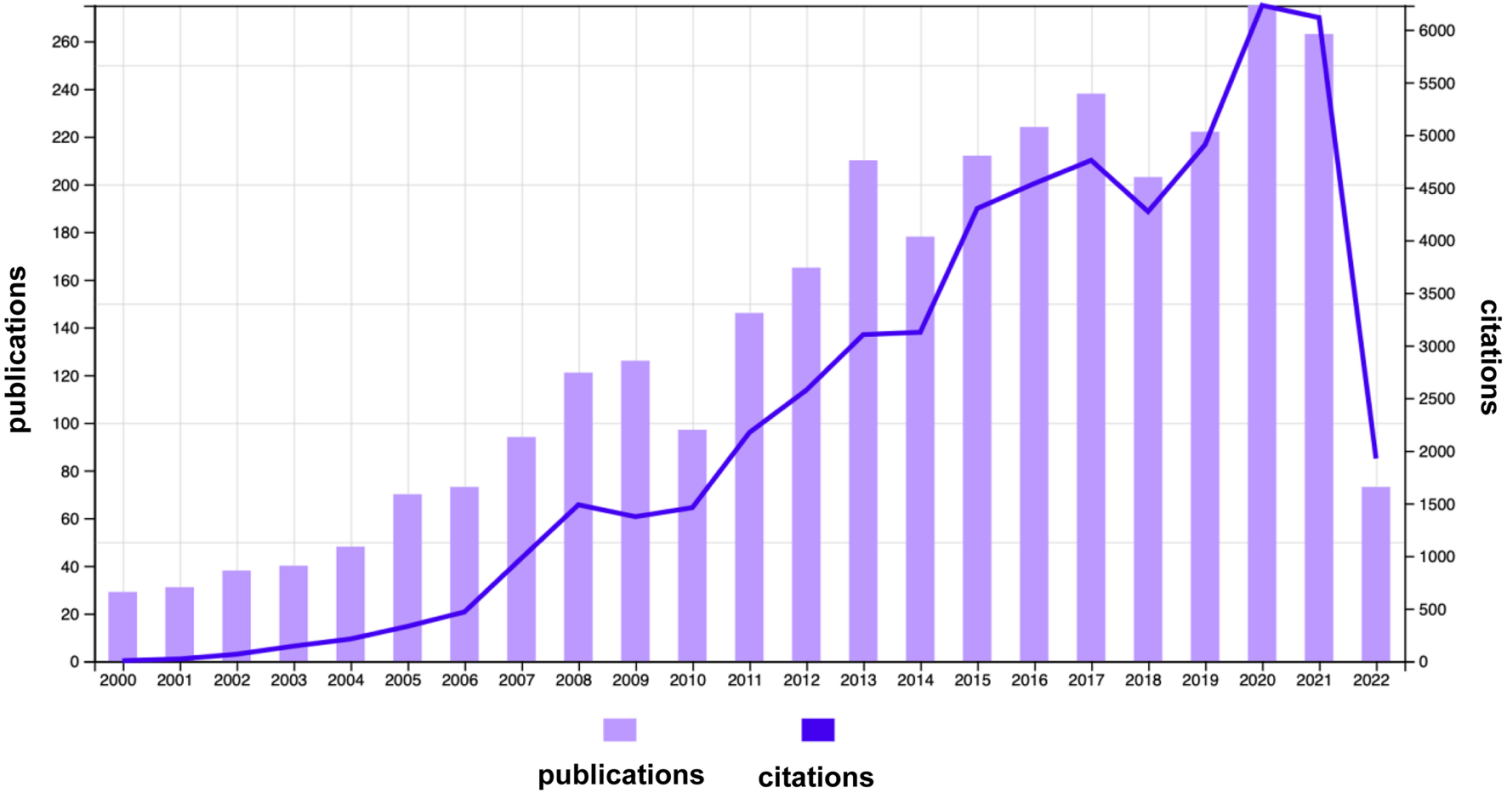
Number of publications and citations each year

A total of 3103 publications related to PNL and urolithiasis were included in the WOS core database, including 2485 original articles (80.1%), 327 reviews (10.5%), 291 conference abstracts, conference proceedings, papers, editorial materials, and other types of literatures. Almost 95.2% of them were written in English. The number of papers published each year is shown in Fig. 1. Among them, the number of articles published each year was relatively average, showing an overall upward trend. This showed that PNL had developed steadily and gradually attracted people’s attention.

### Bibliometric analysis of the keywords

The final analysis was for the keywords provided by the author and those that appeared more than five times in the WOS core database. 278 out of 2533 keywords met the threshold.

**Fig. 2:**
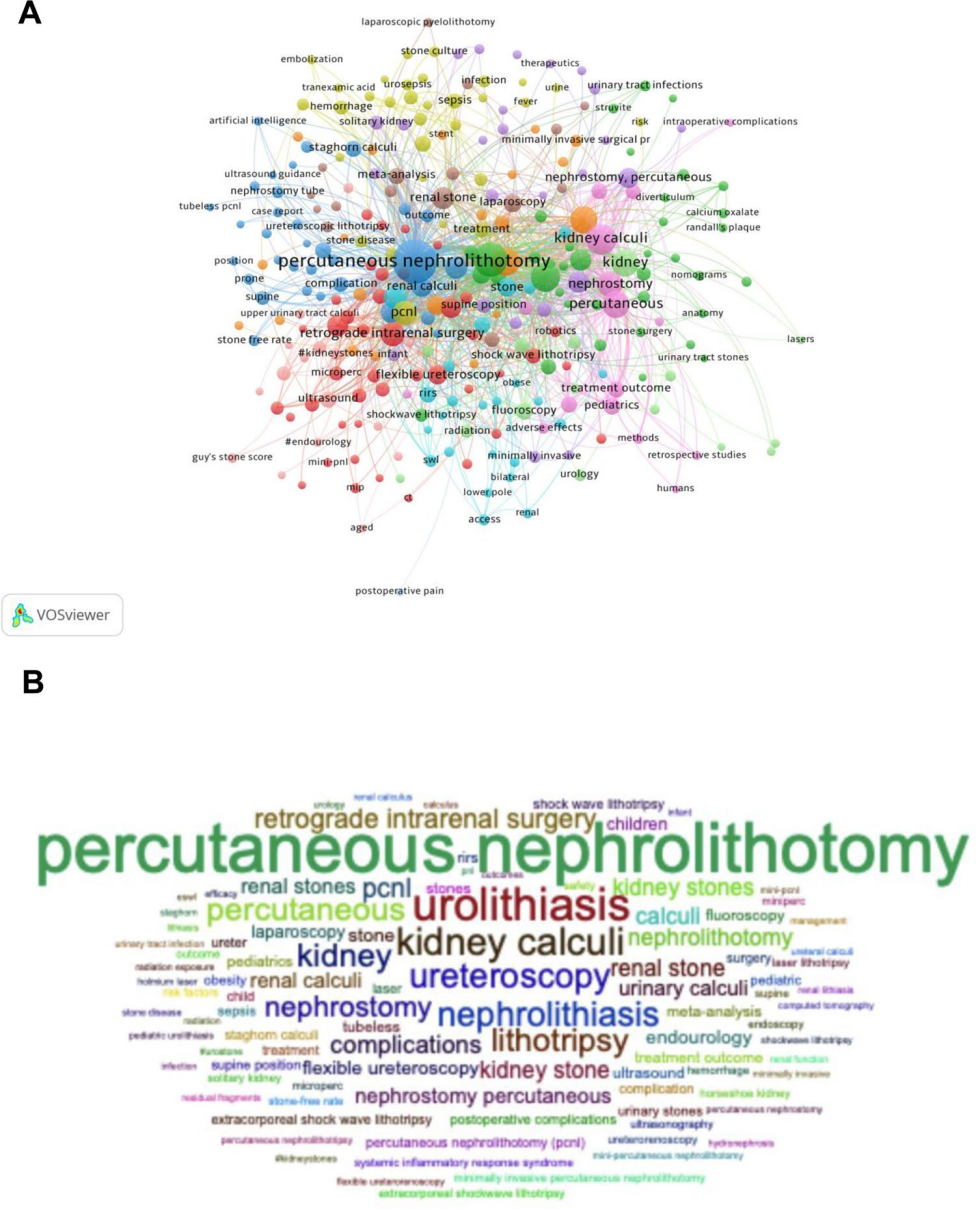
Bibliometric analysis network of the keywords. **A** Bibliometric network of keywords. **B** Word cloud according to the occurrence of keywords

The most common keywords were “percutaneous nephrolithotomy” (total link strength 1816) and “urolithiasis” (total link strength 956), which were closely related to “ureteroscopy” (Fig. 2A).

We created a word cloud chart using the keywords that appeared more than 10 times. The results showed that “percutaneous nephrolithotomy” was the most frequent keyword, followed by “urolithiasis”, “kidney calculi”, “ureteroscopy”, and “nephrolithiasis” (Fig. 2B).

### Bibliometric analysis of the citations and publications

**Fig. 3:**
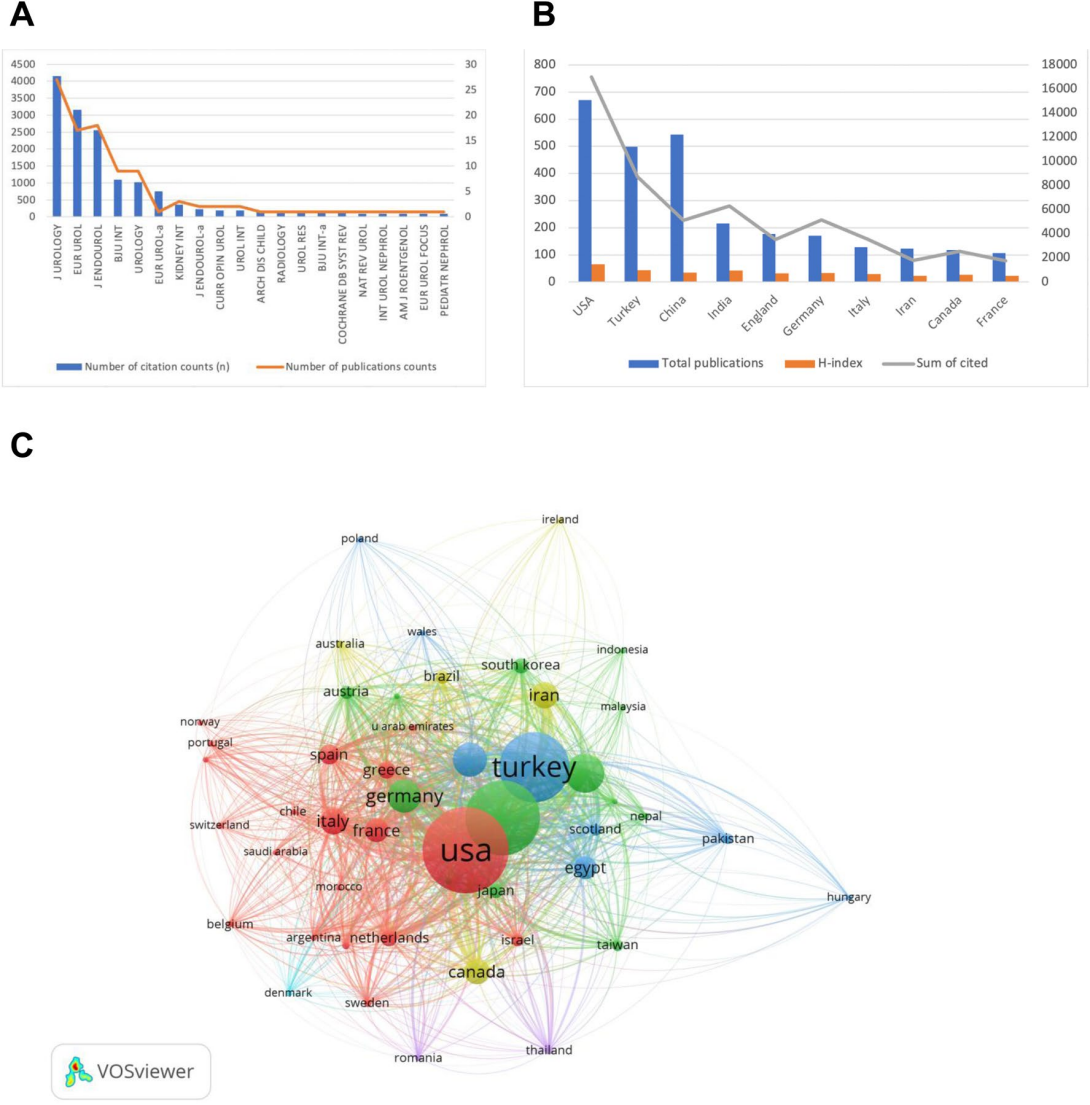
Bibliometric analysis of the citations and publications. **A** Country ranking chart based on total cited. **B** Bibliometric analysis network according to the countries. **C** The publications, H-index, and citations of the top 10 countries

The top 10 most cited articles are listed in Table 1. A total of 276 journals publishing articles related to PNL and urolithiasis were included in the WOS core database, and 5 of them published more than 5 articles. “Journal of Urology” had the largest number of articles (27) with an impact factor of 7.45 (Fig. 3A).

**Table 1:** Top 10 most cited publications

Title	Year	Journal	Dol	Total citations	TC per year	Normalized TC
Turk C	2016	EUR Urol-a	10.1016/j.eururo.2015.07.041	750	107.1429	49.8664
Preminger GM	2005	J Urology	10.1097/01.ju.0000161171.67806.2a	619	34.3889	11.1546
Michel MS	2007	EUR Urol	10.1016/j.eururo.2006.10.020	577	36.0625	15.8174
Pearle MS	2005	J Urology	10.1097/01.ju.0000152082.14384.d7	560	31.1111	10.0914
de la Rosette JJMCH	2011	J Endourol	10.1089/end.2010.0424	473	39.4167	16.5408
Turk C	2016	EUR Urol	10.1016/j.eururo.2015.07.040	344	49.1429	22.8721
Kukreja R	2004	J Endourol	10.1089/end.2004.18.715	320	16.8421	7.5442
Tefekli A	2008	EUR Urol	10.1016/j.eururo.2007.06.049	307	20.4667	10.1912
de la Rosette JJMCH	2012	EUR Urol	10.1016/j.eururo.2012.03.055	213	19.3636	9.9702
Seitz C	2012	EUR Urol	10.1016/j.eururo.2011.09.016	196	17.8182	9.1745

The United States published the most articles (671), followed by Turkey (499) and China (544). The most cited country was the United States (16,987), followed by Turkey (8733) and India (6308). The United States, Turkey, and India had the highest H-index at 65, 44, and 43, respectively. It was worth noting that although the total number of publications in China was more than twice that of India, both the total number of citations and H-index were lower than those of India (Fig. 3B, C).

### Bibliometric analysis of the co-authorship

**Fig. 4:**
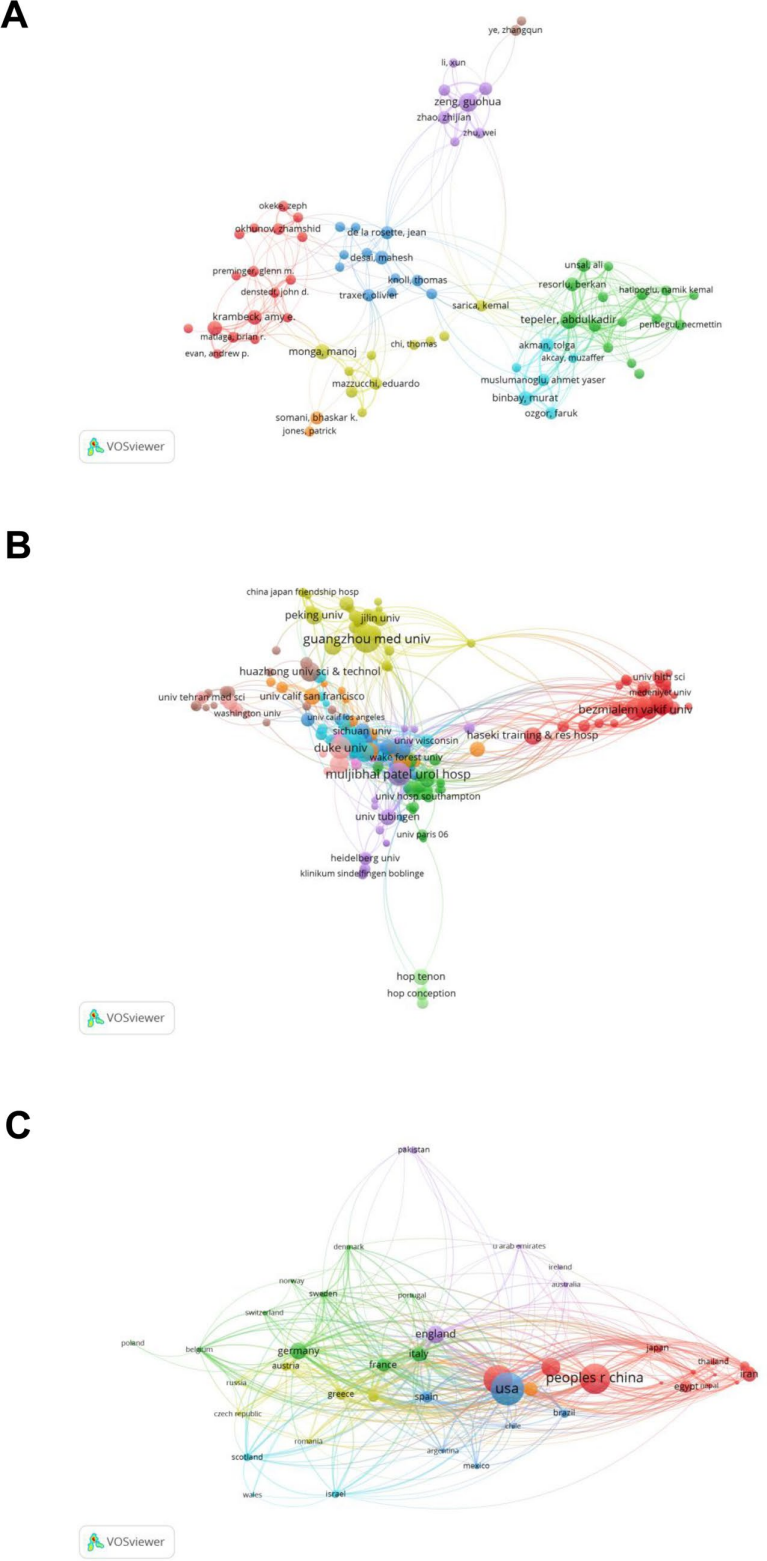
Bibliometric analysis of the co-authorship. **A** Bibliometric analysis network according to the researchers. **B** Bibliometric analysis network according to the institutions. **C** Bibliometric co-authorship analysis network according to the countries

A total of 10,096 researchers published articles on PNL and urolithiasis. During the construction of BNT, only authors with at least five articles were presented, and the total number of authors who met this threshold was 572. The specific amount of publications and citations of the top 10 authors are shown in Table 2. Among them, Zeng Guohua from the First Affiliated Hospital of Guangzhou Medical University published the most articles (62, H-index = 20), and De la Rosette JJMCH from the Department of Urol of Istanbul Medipol University was cited the most (29, H-index = 21) (Fig. 4A).

**Table 2:** Top 10 most productive authors

Author	Documents	Citations
Zeng, Guohua	62	1022
Tepeler, Abdulkadir	54	1070
Monga, Manoj	39	710
Lingeman, James E	38	863
Krambeck, Amy E	38	521
Binbay, Murat	36	799
Unsal, Ali	34	886
Akman, Tolga	33	864
Resorlu, Berkan	33	795
Desai, Mahesh	32	1613

According to the search results, 2324 organizations published relevant articles, of which 262 institutions published more than five papers. Guangzhou Medical University published 76 articles, cited 979 times. It was most closely associated with Istanbul Medipol University, with a connection strength of 4. They studied the use of PNL in urolithiasis (Fig. 4B).

The United States had 40 collaborators with a total link strength of 385 and 671 publications. The main partners of the United States were Canada, China, and Italy with the link strength of 46, 39, and 36, respectively (Fig. 4C).

### Bibliometric analysis of themes and trend topics

**Fig. 5:**
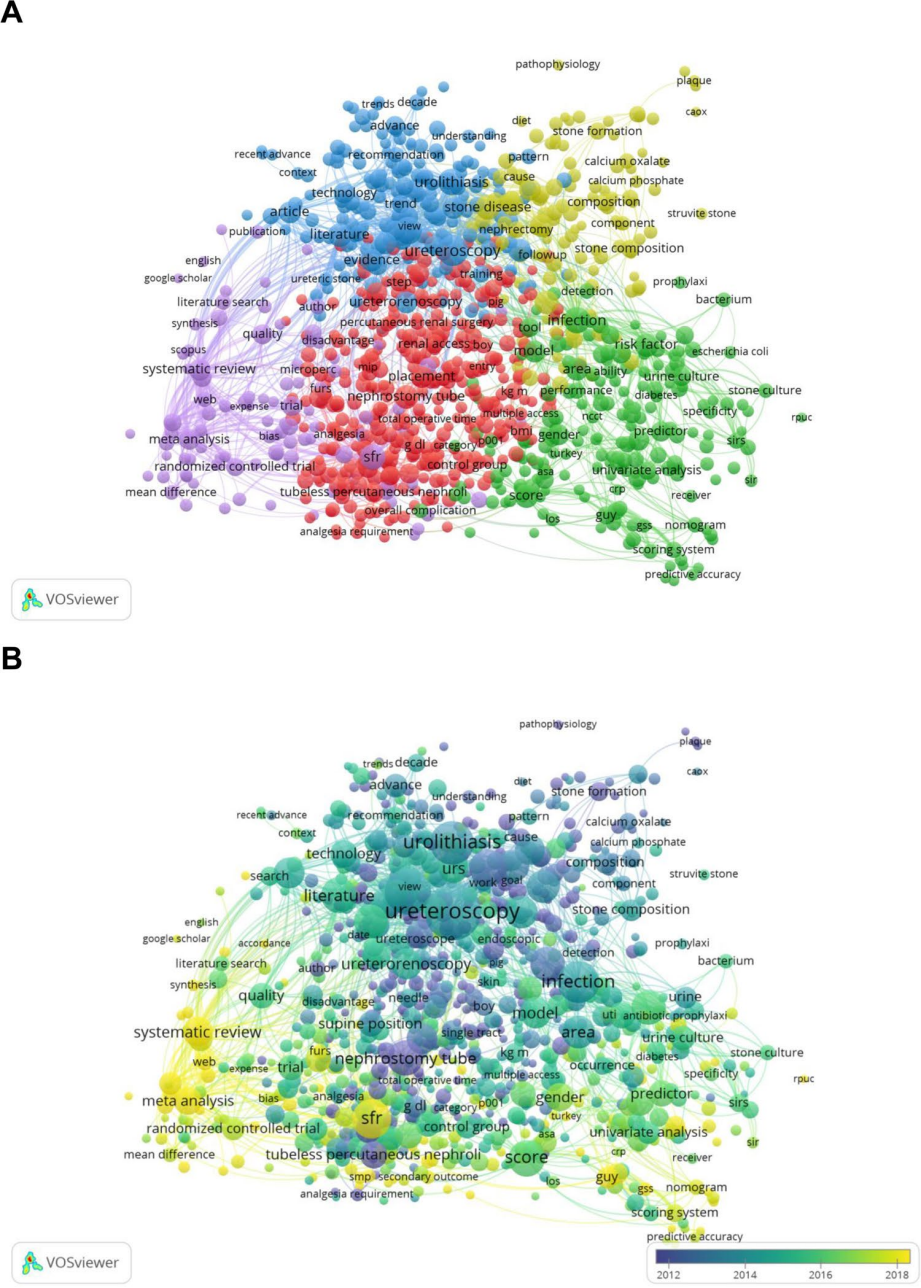
Bibliometric analysis of themes and trend topics. **A** Network visualization of bibliometric analysis of themes and trend topics. **B** Overlay visualization of bibliometric analysis of themes and trend topics

As shown in Fig. 5A, we found five topic clusters. Cluster 1 (red) referred to specific application methods and means. Cluster 2 (green) involved risk factors for urolithiasis. Cluster 3 (blue) dealed with the development of a treatment for urolithiasis. Cluster 4 (yellow) was about the characteristics, composition, and properties of the stones. Cluster 5 (purple) was evaluated for efficacy. Figure 5B shows the trending topics by keywords, with the time period automatically set by VOSviewer to 2012–2018. Indicators showed that current publications changed from purple to yellow.

## Discussion

In this study, we analyzed 3103 publications about PNL and urolithiasis indexed in WOS core database. Through a co-analysis of publication outputs, we knew that research on PNL was maturing. We found that the number of publications was almost the same each year, and there were no major fluctuations, which meant that studies on PNL were growing at a steady rate. Most of these publications were written in English, probably because English-speaking countries dominated this field. In the keywords analysis, the results showed that the most frequent keywords were the applications of PNL, the treatments of urolithiasis, and the complications of PNL, which met our expectations.

Based on publications and citation analysis, we got the top 10 most-cited articles. At the top of the list were two important guidelines [12, 18]. Moreover, 5 of the top 10 articles were about the complications of PNL. PNL can cause bleeding, urine extravasation, and fever, while damage to the colon or pleura was rare [19, 20]. Hemorrhage was one of the most important complications of PNL. Surgical complications (*P* < 0.0001), mature nephrostomy (*P* < 0.0001), operative time (*P* < 0.0001), approach guidance method (fluoroscopy vs ultrasound) (*P* = 0.0001), expansion method (*P* = 0.0001), multiple (> or = 2) bundles (*P* = 0.003), bundle size (*P* = 0.001), renal solid thickness (*P* = 0.05), and diabetes mellitus (*P* = 0.05) were important factors to predict blood loss [21, 22]. Christian Seitz et al. [23] discussed the incidence, prevention, and management of postoperative complications of PNL in more details. A categorization method (Clavien score) for the evaluation of postoperative complications of PNL was established and validated, with high validity for the complications on duration of stay but low reliability for the evaluation of minor complications [24, 25]. A large number of studies on complications proved that PNL was mature and in the stage of self-optimization and improvement. It was worth noting that one of the top 10 articles discussed the relationship between PNL, urolithiasis, and socioeconomic burden. Although the research had a long history and had little reference value for the present, it can still reflect the positive impact of PNL on the social economy in the process of development [26]. Further study of postoperative complications of PNL showed that PNL was widely used and mature. The journals that published the most articles on this topic were *Journal of Urology*, *European Urology*, and *Journal of Endourology*. Among the top 20 journals that published articles of this field, publications accounted for more than half of the total, which may indicate that articles in the field were published intensively.

From the perspective of country analysis, the United States was the most developed country in this field, with the largest number of articles and the highest H-index, indicating that the United States led not only in quantity but also in quality in this field. Therefore, it is urgent to improve the research quality of Chinese scholars. Although there were also a large number of articles published in China, its H-index was relatively low, which may indicate that Chinese scholars value quantity more than quality. Moreover, it is worth noting that although the total number of papers published in India was relatively small, its H-index was high, indicating that the research quality of Indian scholars was relatively high.

From the author’s analysis, we can also see that the one who published the most articles was a Chinese scholar with a relatively low H-index. A Turkish scholar with the highest H-index published less than half of that Chinese scholars, which further confirmed that Chinese scholars paid more attention to the quantity of research rather than the quality. In terms of cooperation between countries, the United States had close cooperation with Canada, China, Italy, and many other countries, because higher research quality brings the United States closer to international cooperation.

In conclusion, this study not only provided urologist with valuable information about PNL and urolithiasis, but also provided a new way for researchers to understand urolithiasis. This manuscript also helped investigators understand research hotspots, collaborating institutions, and research frontiers.

## Data Availability

Not applicable.

## Abbreviations

PNL: Percutaneous nephrolithotomy
ESWL: Extracorporeal shock wave lithotripsy
WOS: Web of science
BNT: Bibliometric network
